# Supplementary material for: AI-Assisted identification of sex-specific patterns in diabetic retinopathy using retinal fundus images
Source: PLoS One. 2025 Aug 7;20(8):e0327305. doi: 10.1371/journal.pone.0327305 (PMC12331106; doi:10.1371/journal.pone.0327305)
Supplement: S1 Table — (PDF) [file pone.0327305.s001.pdf]

Table S1. Composition of the CNN Training set. NPDR: Non-proliferative DR.

**CNN Training Set**				
		Female	Male	Total
N		1043	1028	2071
Age (M ± SD)		51.95 ± 11.36	50.40 ± 9.97	51.18 ± 10.72
Ethnicity (N)				
	Latin American	778	704	1482
	Caucasian	95	166	261
	Multi-racial	56	37	93
	Asian	40	38	78
	African Descent	35	46	81
	Other	27	17	44
	Native American	6	10	16
	Indian Subcontinent Origin	2	8	10
Severity of DR (N)				
	Moderate NPDR	500	569	1069
	Mild NPDR	463	378	841
	Severe NPDR	41	58	99
	Proliferative NPDR	39	23	62
HbA1c (M ± SD)		9.01 ± 2.29	8.97 ± 4.47	8.99 ± 3.55
