# Supplementary material for: AI-Assisted identification of sex-specific patterns in diabetic retinopathy using retinal fundus images
Source: PLoS One. 2025 Aug 7;20(8):e0327305. doi: 10.1371/journal.pone.0327305 (PMC12331106; doi:10.1371/journal.pone.0327305)
Supplement: S2 Table — (PDF) [file pone.0327305.s002.pdf]

Table S2. Composition of the CNN Validation set. NPDR: Non-proliferative DR.

CNN Validation Set				
		Female	Male	Total
N		224	224	448
Age (M $\pm$ SD)		53.10 ± 10.54	51.75 ± 10.23	52.42 ± 10.41
Ethnicity (N)				
	Latin American	172	159	331
	Caucasian	21	33	54
	Multi-racial	10	12	22
	Asian	8	7	15
	African Descent	5	5	10
	Other	0	4	4
	Native American	5	2	7
	Indian Subcontinent Origin	2	2	4
Severity of DR (N)				
	Moderate NPDR	94	103	197
	Mild NPDR	111	102	213
	Severe NPDR	10	9	19
	Proliferative NPDR	9	10	19
HbA1c (M $\pm$ SD)		8.87 ± 5.80	8.80 ± 2.17	8.83 ± 4.38
