# Supplementary material for: AI-Assisted identification of sex-specific patterns in diabetic retinopathy using retinal fundus images
Source: PLoS One. 2025 Aug 7;20(8):e0327305. doi: 10.1371/journal.pone.0327305 (PMC12331106; doi:10.1371/journal.pone.0327305)
Supplement: S3 Table — (PDF) [file pone.0327305.s003.pdf]

Table S3. Composition of the CNN Test set. NPDR: Non-proliferative DR.

**CNN Test Set**				
		Female	Male	Total
N		224	224	448
Age (M $\pm$ SD)		53.43 ± 10.04	50.89 ± 11.15	52.16 ± 10.69
Ethnicity (N)				
	Latin American	168	148	316
	Caucasian	20	41	61
	Multi-racial	13	9	22
	Asian	4	8	12
	African Descent	6	9	15
	Other	7	5	12
	Native American	3	4	7
	Indian Subcontinent Origin	3	0	3
Severity of DR (N)				
	Moderate NPDR	102	122	224
	Mild NPDR	105	88	193
	Severe NPDR	11	8	19
	Proliferative NPDR	6	6	12
HbA1c (M $\pm$ SD)		8.77 ± 1.99	8.79 ± 2.11	8.78 ± 2.05
