# Supplementary figures and images for: AI-Assisted identification of sex-specific patterns in diabetic retinopathy using retinal fundus images
Source: PLoS One. 2025 Aug 7;20(8):e0327305. doi: 10.1371/journal.pone.0327305 (PMC12331106; doi:10.1371/journal.pone.0327305)

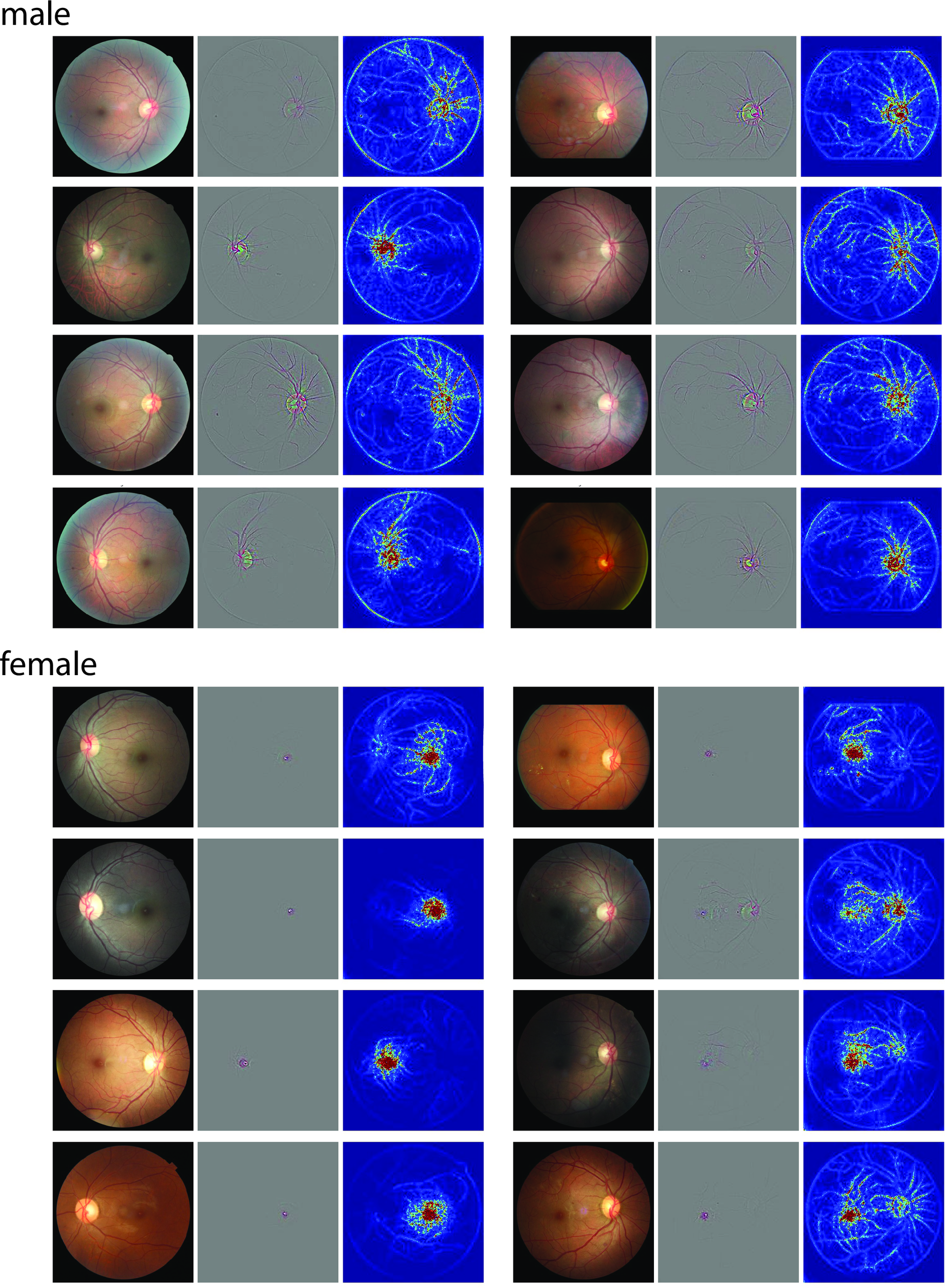

Supplement: S1 Fig — Sixteen images were randomly chosen to demonstrate the consistency of the saliency map results. (TIF) [file pone.0327305.s004.tif]

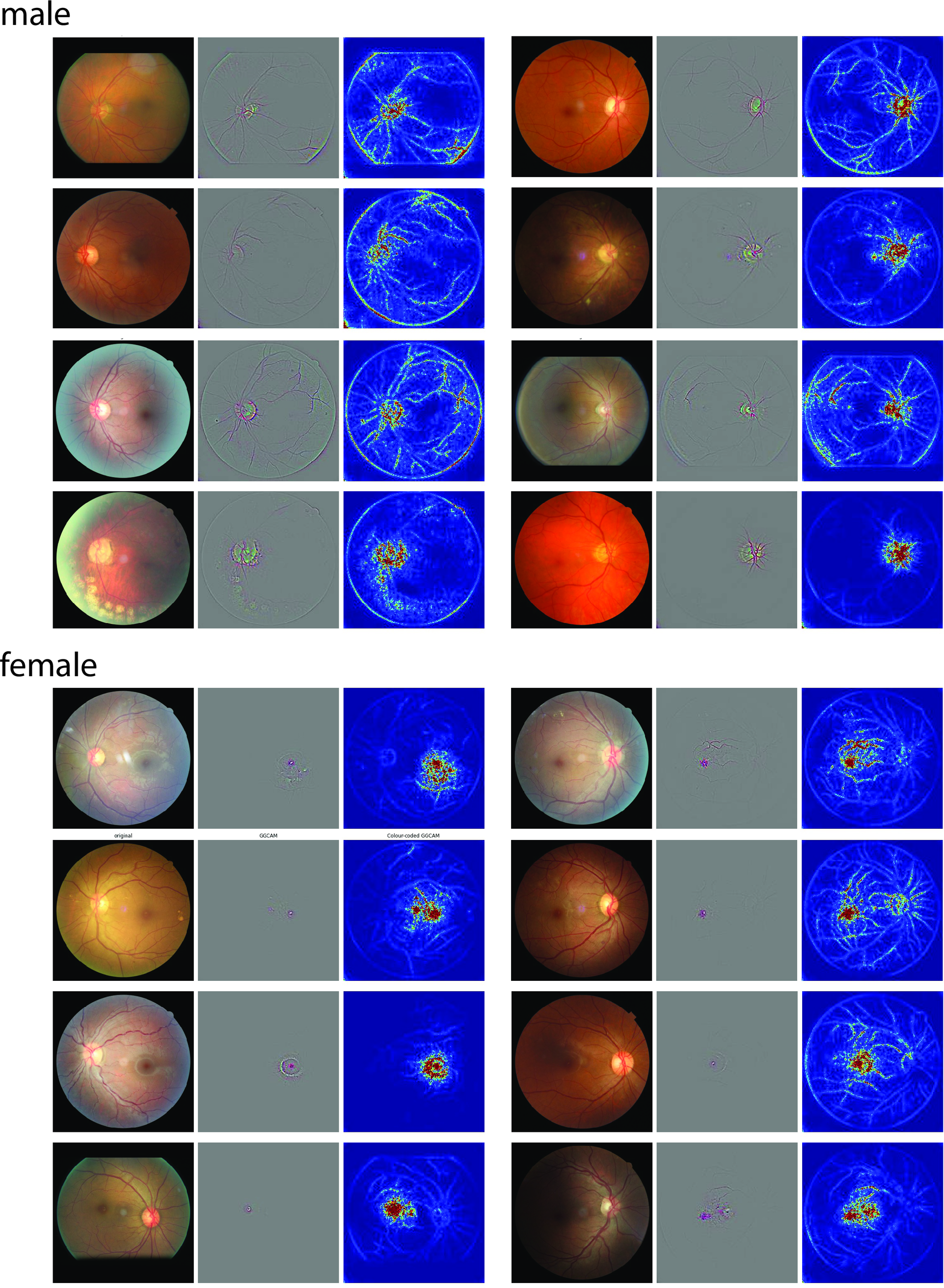

Supplement: S2 Fig — Sixteen images were randomly chosen to demonstrate the consistency of the saliency map results. (TIF) [file pone.0327305.s005.tif]
