# Supplementary material for: AI-Assisted identification of sex-specific patterns in diabetic retinopathy using retinal fundus images
Source: PLoS One. 2025 Aug 7;20(8):e0327305. doi: 10.1371/journal.pone.0327305 (PMC12331106; doi:10.1371/journal.pone.0327305)
Supplement: Attachment — Submitted filename: ResponseToReviews.pdf [file pone.0327305.s006.pdf]

Dear PLOS One Editorial Board,

We have been asked to "provide a point-by-point response to the reviewers and any Academic Editor’s comments in a separate document". Our manuscript was not sent out for peer-review, therefore there are no reviewer comments that we can respond to. Below, we respond to the Academic Editor's comments in a point-by-point fashion.

Academic Editor's comments:

The manuscript is concerned with the application of the VGG16 architecture for analysis of fundus images in the context of gender-specific diabetic retinopathy, coupled with Guided Grad-CAM to support the interpretability of the results, through the analysis of the associated feature maps. The application area of this research is interesting, however, there are a few major concerns.

- Overall, beyond the application area, there is a lack of novelty as both techniques are very well known in the state-of-art.

Our Response: Our study employs well-established, state-of-the-art machine learning techniques to analyze retinal fundus images, producing original results that could significantly impact the diagnosis and management of diabetic retinopathy. This is the novelty of our contribution. In fact, it is better practice not to test new algorithms or techniques on novel applications. We do not claim that this is a computer science paper introducing a new algorithm. Furthermore, PLOS ONE is not a computer science journal. Our study is novel for its application area and for the significance of our results.

For these reasons, our paper should not have been rejected (without peer review) based on the rationale that "there is a lack of novelty".

- VGG16 is relatively shallow, which may limit its ability to capture the complex patterns and subtle features that are critical for detecting early signs of diabetic retinopathy, especially when factoring in gender-specific variations. Moreover, it employs a series of max-pooling layers that downsample the input images progressively. This aggressive downsampling can lead to a loss of fine spatial details that are crucial for detecting small lesions, microaneurysms, or subtle gender-specific retinal features associated with diabetic retinopathy.

Although VGG16 is considered relatively shallow compared to more recent architectures with similar performance, it is still a deep learning model and with its 16 layers, it is not "shallow" in the traditional sense. It remains one of the most popular architectures in image processing. In fact, VGG16 [1-4], VGG19 ([5] published in PLOS One, cited 283 times) and even shallower architectures like AlexNet [6] have been successfully applied to retinal fundus images for automated classification of ocular disease. It is not always a foregone conclusion that the deepest model is the best choice for all applications. Often, simpler models provide better interpretability, which is why we opted for VGG16. Given its strong performance relative to our training set size, VGG16 was the preferred model for this task.

Furthermore, in a recent study published in PNAS Nexus [7], we identified five subtle retinal markers that differentiate sex in fundus images, and successfully trained ophthalmologists to use these markers, all identified using VGG16, to recognize sex from fundus images—something they could not do prior to training (which was also shown in [7]). This strong evidence clearly refutes the notion that VGG16 is incapable of detecting subtle retinal signals, as the model played a key role in this discovery.

- If VGG16 misclassifies a fundus image due to its inherent limitations (e.g., poor handling of small-scale features), Guided Grad-CAM will still produce a heatmap, but the highlighted areas may be misleading, pointing to regions that have no relation to diabetic retinopathy or gender-specific features. This is problematic in medical contexts, where misinterpretation can affect diagnosis and treatment decisions.

As clearly specified in our manuscript, we did not use misclassified images to generate heatmaps. We only used accurately classified images for this purpose. Please see lines 131-138, also included below:

Model Interpretation

Figures 1 and 2 depict sample saliency-maps for eight patients—two male and two female for each model classes—using the ImageNet and sex-trained models, respectively. The original fundus images, Guided Grad-CAM outputs, and the color-coded saliency maps are shown in the left, middle, and right panels, respectively. The images are selected as correctly classified examples from the test set. These sample maps show that the network focuses on distinct anatomical regions of the retina for male and female DR patients, with the highlighted in male eyes consistently differing from those in female eyes.

- Importantly, the results of Guided Grad-CAM are affected by the choice of the CNN structure. Since Guided Grad-CAM generates visual explanations by analyzing gradients flowing through the network and highlighting important regions based on the activation maps of the convolutional layers, the CNN architecture plays a critical role in shaping the output. Instead, additional experiments with a vareity of CNN architectures are needed, or alternatively, to consider approaches such as attention mechanisms, self-supervised learning and integrated gradients.

The primary result of interest in the submitted work is that when the model is held constant (VGG16), and the task identical (binary sex classification), the Grad-Cam results change significantly and meaningfully when the images were of individuals with diabetic retinopathy compared to those of healthy individuals (which were reported in [7]). While other models and machine learning techniques can certainly be applied, there must be proper justification before exploring countless alternatives. We respectfully ask peer-reviewers to weigh in on this, rather than the paper being rejected outright.

Best regards,
Ipek Oruc, on behalf of all authors

References

[1] Ting, D. S. W., Cheung, C. Y. L., Lim, G., Tan, G. S. W., Quang, N. D., Gan, A., ... & Wong, T. Y. (2017). Development and validation of a deep learning system for diabetic retinopathy and related eye diseases using retinal images from multiethnic populations with diabetes. *Jama*, *318*(22), 2211-2223. (cited 1991 times)

[2] Da Rocha, D. A., Ferreira, F. M. F., & Peixoto, Z. M. A. (2022). Diabetic retinopathy classification using VGG16 neural network. *Research on Biomedical Engineering*, *38*(2), 761-772.

[3] Arora, A., Gupta, S., Singh, S., & Dubey, J. (2022, July). Eye disease detection using transfer learning on vgg16. In *Proceedings of Third International Conference on Computing, Communications, and Cyber-Security: IC4S 2021* (pp. 527-536). Singapore: Springer Nature Singapore.
[4] Rim, T. H., Lee, G., Kim, Y., Tham, Y. C., Lee, C. J., Baik, S. J., ... & Cheng, C. Y. (2020). Prediction of systemic biomarkers from retinal photographs: development and validation of deep-learning algorithms. *The Lancet Digital Health*, *2*(10), e526-e536.

[5] Choi, J. Y., Yoo, T. K., Seo, J. G., Kwak, J., Um, T. T., & Rim, T. H. (2017). Multi-categorical deep learning neural network to classify retinal images: A pilot study employing small database. *PloS one*, *12*(11), e0187336. (cited 283 times)

[6] Burlina, P. M., Joshi, N., Pekala, M., Pacheco, K. D., Freund, D. E., & Bressler, N. M. (2017). Automated grading of age-related macular degeneration from color fundus images using deep convolutional neural networks. *JAMA ophthalmology*, *135*(11), 1170-1176. (cited 636 times)

[7] Delavari, P., Ozturan, G., Yuan, L., Yilmaz, Ö., & Oruc, I. (2023). Artificial intelligence, explainability, and the scientific method: A proof-of-concept study on novel retinal biomarker discovery. *PNAS nexus*, *2*(9), pgad290.
