# Supplementary material for: AI-Assisted identification of sex-specific patterns in diabetic retinopathy using retinal fundus images
Source: PLoS One. 2025 Aug 7;20(8):e0327305. doi: 10.1371/journal.pone.0327305 (PMC12331106; doi:10.1371/journal.pone.0327305)
Supplement: Attachment — Submitted filename: ResponseLetter_Delavari_etal_final.pdf [file pone.0327305.s008.pdf]

Dear Dr. Howard,

Thank you for your editorial work on our manuscript for PLOS ONE. We also thank the two reviewers for their thoughtful and clear feedback. Below we describe, in a point-by-point fashion, how we have addressed the journal requirements as well as each issue raised by the reviewers. These revisions resulted in several text changes in the Introduction, Methods, and Discussion sections, and Supporting Materials. We hope that, with these revisions, our manuscript is now acceptable to be published in PLOS ONE.

Journal requirements:

When submitting your revision, we need you to address these additional requirements.

1. Please ensure that your manuscript meets PLOS ONE's style requirements, including those for file naming. The PLOS ONE style templates can be found at https://journals.plos.org/plosone/s/file?id=wjVg/PLOSOne_formatting_sample_main_body.pdf and https://journals.plos.org/plosone/s/file?id=ba62/PLOSOne_formatting_sample_title_authors_affiliations.pdf

We have now revised our manuscript to ensure PLOS ONE's style requirements are met, including those for file naming.

2. Please note that PLOS ONE has specific guidelines on code sharing for submissions in which author-generated code underpins the findings in the manuscript. In these cases, we expect all author-generated code to be made available without restrictions upon publication of the work. Please review our guidelines at https://journals.plos.org/plosone/s/materials-and-software-sharing#loc-sharing-code and ensure that your code is shared in a way that follows best practice and facilitates reproducibility and reuse.

We have made the code used to generate the results in the submitted manuscript publicly available in the following GitHub repository: *https://github.com/Fundus-AI/DR_sex_difference*. The repository link has been included at the end of the Methods section.

3. Please expand the acronym "NSERC" (as indicated in your financial disclosure) so that it states the name of your funders in full.
This information should be included in your cover letter; we will change the online submission form on your behalf.

We have now included in our cover letter that our funder NSERC is 'Natural Sciences and Engineering Research Council of Canada'.

4. We note that you have indicated that there are restrictions to data sharing for this study. For studies involving human research participant data or other sensitive data, we encourage authors to share de-identified or anonymized data. However, when data cannot be publicly shared for ethical reasons, we allow authors to make their data sets available upon request. For information on unacceptable data access restrictions, please see http://journals.plos.org/plosone/s/data-availability#loc-unacceptable-data-access-restrictions.

Before we proceed with your manuscript, please address the following prompts:

a) If there are ethical or legal restrictions on sharing a de-identified data set, please explain them in detail (e.g., data contain potentially identifying or sensitive patient information, data are owned by a third-party organization, etc.) and who has imposed them (e.g., a Research Ethics Committee or Institutional Review

Board, etc.). Please also provide contact information for a data access committee, ethics committee, or other institutional body to which data requests may be sent.

Data cannot be shared publicly because data are owned by a third party and we do not have permission to share the data. The data underlying the results presented in the study are available from EyePACS.

https://eyepacs.com/

contact@eyepacs.org

b) If there are no restrictions, please upload the minimal anonymized data set necessary to replicate your study findings to a stable, public repository and provide us with the relevant URLs, DOIs, or accession numbers. Please see http://www.bmj.com/content/340/bmj.c181.long for guidelines on how to de-identify and prepare clinical data for publication. For a list of recommended repositories, please see https://journals.plos.org/plosone/s/recommended-repositories. You also have the option of uploading the data as Supporting Information files, but we would recommend depositing data directly to a data repository if possible.

Please update your Data Availability statement in the submission form accordingly.

Additional Editor Comments (if provided):

[Note: HTML markup is below. Please do not edit.]

Reviewers' comments:

Reviewer's Responses to Questions

Comments to the Author

1. If the authors have adequately addressed your comments raised in a previous round of review and you feel that this manuscript is now acceptable for publication, you may indicate that here to bypass the “Comments to the Author” section, enter your conflict of interest statement in the “Confidential to Editor” section, and submit your "Accept" recommendation.

Reviewer #1: All comments have been addressed

Reviewer #2: (No Response)

---

2. Is the manuscript technically sound, and do the data support the conclusions?

The manuscript must describe a technically sound piece of scientific research with data that supports the conclusions. Experiments must have been conducted rigorously, with appropriate controls, replication, and sample sizes. The conclusions must be drawn appropriately based on the data presented.

Reviewer #1: Yes

Reviewer #2: Partly

---

3. Has the statistical analysis been performed appropriately and rigorously?

Reviewer #1: Yes

Reviewer #2: No

---

4. Have the authors made all data underlying the findings in their manuscript fully available?

The PLOS Data policy requires authors to make all data underlying the findings described in their manuscript fully available without restriction, with rare exception (please refer to the Data Availability Statement in the manuscript PDF file). The data should be provided as part of the manuscript or its supporting information, or deposited to a public repository. For example, in addition to summary statistics, the data points behind means, medians and variance measures should be available. If there are restrictions on publicly sharing data—e.g. participant privacy or use of data from a third party—those must be specified.

Reviewer #1: No

Reviewer #2: Yes

---

5. Is the manuscript presented in an intelligible fashion and written in standard English?

PLOS ONE does not copyedit accepted manuscripts, so the language in submitted articles must be clear, correct, and unambiguous. Any typographical or grammatical errors should be corrected at revision, so please note any specific errors here.

Reviewer #1: Yes

Reviewer #2: Yes

6. Review Comments to the Author

Please use the space provided to explain your answers to the questions above. You may also include additional comments for the author, including concerns about dual publication, research ethics, or publication ethics. (Please upload your review as an attachment if it exceeds 20,000 characters)

Reviewer #1:

• The rationale for identifying sex-specific patterns in diabetic retinopathy is not well-justified. The authors should provide a stronger explanation of the clinical relevance and potential impact of their findings. • It is unclear how this approach contributes to improving diagnosis or treatment strategies compared to traditional methods.

We thank the reviewer for highlighting the need to better justify the rationale and clinical relevance of our findings. We address the first two points above together as these are closely related.

Our results suggest that DR may manifest differently in males and females; therefore, understanding these distinctions could lead to earlier and more accurate diagnoses. Although evidence for sex differences in DR is currently limited, there is evidence that diabetes, as well as complications of diabetes, such as diabetic kidney disease, show sex differences (as cited in the paper). This suggests that DR, as another diabetes-related condition, may also exhibit sex-specific patterns due to distinct physiological responses in males and females. Recognizing these differences could enable earlier, more precise diagnoses, leading to timely treatment as well as tailored management strategies that account for the unique presentation of DR in both sexes.

To address the reviewer's comment, we have added the following new text to the body of the manuscript

Introduction

lines 9-19

"Similarly, while diabetes is more prevalent among men [11], women are diagnosed at older age, greater disease severity, and higher body fat mass than men [12–15]. In addition, growing evidence suggests that diabetic complications manifest differently in women and men [16, 17]. For instance, some studies indicate that women and girls may be at greater risk of developing diabetic kidney disease compared to age-matched men and boys [18, 19]. Computational modeling predictions further support sex-specific differences in diabetes-induced changes in kidney function, highlighting distinct physiological responses in males and females [20]. Elucidating specific fine-grained diagnostic markers for men and women would enhance diagnostic precision and enable earlier detection of disease across diverse populations.

lines 22-28

"Delays to the diagnosis of DR poses significant risks, including progression to vision-threatening stages and systemic complications. Identifying novel sex-specific markers would likely enhance the diagnostic accuracy of DR, enabling earlier and more precise detection of the disease. There is some evidence in the literature that sex and gender play a role in how this condition develops, manifests and progresses [17–21]. However, aside from considerations during pregnancy, sex is not factored into the diagnosis, management or treatment of DR."

Discussion

lines 211-254

Differences in the manifestation of DR between males and females have not been extensively investigated, and our findings are among the first to suggest that such differences may exist. Failing to account for these differences in diagnostic approaches may lead to higher false positive and false negative rates. For example, diagnostic algorithms or clinical assessments that consider all symptoms (i.e., the union of symptoms in both sexes) may result in increased false positives, while those that focus only on common symptoms could overlook sex-specific features, increasing false negatives. By identifying and incorporating sex-specific features, diagnostic methods could be refined to reduce both types of errors, enable more accurate decisions, and mitigate the risks of delayed diagnosis by facilitating earlier detection of DR onset.

The risks of delayed diagnosis of DR, the leading cause of blindness among persons of working age in the industrialized world, are substantial and include progression to severe vision-threatening stages and systemic complications [44]. Early diagnosis alerts patients to the systemic onset of microvascular damage, signaling that their diabetes has progressed to a critical stage [45]. This awareness often prompts patients to adopt stricter glycemic control, especially for those who have not yet prioritized diabetes management. DR is also strongly correlated with diabetic kidney disease (DKD), and an early diagnosis of DR encourages closer monitoring of kidney function, including routine blood work, which can help detect and address kidney complications before they progress. Improved diabetes management and regular monitoring can reduce the risk of future complications, including cardiovascular disease, neuropathy, and kidney failure [46].

From an ocular perspective, early diagnosis is particularly beneficial for patients in the mild and moderate stages of DR, as the disease often progresses silently without symptoms in its early stages, delaying intervention. Regular follow-ups and monitoring at these stages provide an opportunity for timely intervention, which can help prevent progression to advanced stages [47]. More importantly, it is particularly crucial to identify and manage high-risk non-proliferative DR patients due to their approximately 50% likelihood of progressing to proliferative DR (PDR) within a year [48]. PDR, the advanced stage of DR, can cause blindness through retinal neovascularization-related complications such as vitreous hemorrhages, tractional retinal detachments, and neovascular glaucoma. At this advanced stage, even with treatment, the likelihood of meaningful visual recovery is significantly reduced [49].

Current treatment options for DR, including pan-retinal photocoagulation (PRP) and intravitreal anti-VEGF and glucocorticosteroids injections, have revolutionized outcomes for DR patients

[47]. Severe vision loss (<20/200) in DR was once common, affecting approximately 50% of patients with PDR in the pre-treatment era [50]. Today, these rates have decreased in patients receiving appropriate treatment. However, the effectiveness of these treatments is closely tied to the stage of DR at the time of intervention. The chance of preventing vision loss or achieving visual recovery diminishes significantly as the disease progresses to advanced stages [51]. Thus, early and more accurate diagnosis not only reduces diagnostic errors but also enables timely intervention, which can prevent the progression of DR and significantly improve patient outcomes.

• The dataset used for training and validation is not described in detail. Critical information such as sample size, class balance, preprocessing steps, and demographic distribution is missing.

We appreciate the reviewer's feedback and have addressed these concerns by adding three additional tables to the supplementary materials. These tables provide detailed summary statistics, including sample sizes, class distributions, demographic information, DR severity levels, and HbA1c levels, separately for the training, validation, and test sets. These tables serve as breakdowns of the information presented in Table 1 of the manuscript.

Regarding preprocessing, we did not perform any specific steps other than cropping the images to a square format to prepare them for input into the CNN model. We have added a sentence to the Methods section to describe this step:

Each fundus image was cropped into a square format by detecting the circular contour of the fundus and centering it within a square with equal height and width.

Additionally, we utilized data augmentation and image transformations to improve the generalizability of the model, as detailed in the "Data Augmentation and Transforms" section of the Methods. Briefly, data augmentation included applying random rotations of the images (uniformly chosen between -10 to +10 degrees) and a horizontal flipping transformation for all right-eye images. The horizontal flipping was specifically designed to address the inherent left-right symmetry of the retina, reducing task-irrelevant variation and ensuring consistent anatomical retinal features across the dataset.

• Include more recent references that are relevant to the topic, such as

• Al-hazaimeh, Obaida M., Ashraf A. Abu-Ein, Nedal M. Tahat, Ma'moun A. Al-Smadi, and Malek M. Al-Nawashi. "Combining Artificial Intelligence and Image Processing for Diagnosing Diabetic Retinopathy in Retinal Fundus Images." International Journal of Online & Biomedical Engineering 18, no. 13 (2022).
• Gharaibeh, Nasr, Obaida M. Al-Hazaimeh, Bassam Al-Naami, and Khalid MO Nahar. "An effective image processing method for detection of diabetic retinopathy diseases from retinal fundus images." International Journal of Signal and Imaging Systems Engineering 11, no. 4 (2018): 206-216.

We thank the reviewer for bringing these publications to our attention. We have now added these references to the Introduction section.

Reviewer #2:

It is a good study with a reasonably good sample size of retinal images, which is age and gender adjusted.

1. While the conclusions mentions that diabetic macular edema (DME) appears to be more common in women and PDR in men as assessed by the AI system, I do not find any information on DME in the tables supporting the conclusion. Can you kindly provide the same?

We thank the reviewer for their observation. We would like to clarify that we have not claimed that diabetic macular edema (DME) is more common in women or that proliferative diabetic retinopathy (PDR) is more common in men. Rather, our AI-based analysis suggests that there may be differences in how diabetic retinopathy (DR) signs manifest in the retina between women and men. Specifically, our findings indicate that the macular region may play a more significant role in detecting DR signs in women, while peripheral regions, particularly around the optic disc and along the vascular arcades, appear to be more relevant in men.

This raises an important hypothesis that women might be at a higher risk for DME, whereas men might be at a higher risk for PDR. It is important to emphasize that saliency maps only highlight the regions attended to by the model when making its predictions. While they indicate which regions contribute to predictions, they do not definitively reveal which specific features or lesions (e.g., neovascularization or macular edema) the model relies on.

We presented this as a potential direction for future research, not as a definitive conclusion. We recognize that further studies are necessary to validate these observations and explore their implications in clinical practice. We have now revised the wording in the conclusions to ensure that our intended meaning is clearly conveyed:

lines 195-210

DR, from its onset at mild non-proliferative stages through moderate and severe non-proliferative DR to PDR and macular edema, manifests with hallmark lesions such as hard exudates, cotton wool spots, microaneurysms, and hemorrhages. Based on the interpretation of our models trained on eyes with DR, it is possible that the manifestation of DR differs between males and females. For instance, saliency maps generated by our model suggest that the macular region may play a more significant role in DR classification for females, whereas peripheral regions, particularly around the optic disc and along the vascular arcades, are more relevant for males. These patterns raise the hypothesis that women might be at a higher risk for macular edema, while men might be at a higher risk for PDR. However, it is important to note that saliency maps only highlight the regions the model attends to when making its predictions; while they inform us about which areas are important for the classification task, they do not definitively indicate which features or lesions, such as neovascularization or macular edema, are being used by the model. Further research is needed to validate this hypothesis; if confirmed, it could have significant implications for the diagnosis, management, and treatment of DR.

2. If the duration of diabetes is available, that information can be added as it is one of the key factors in DR irrespective of the gender.

We thank the reviewer for their suggestion. Unfortunately, accurate data on the duration of diabetes is not available to us. However, even in the presence of diabetes duration data, we

believe that DR severity levels provide a more direct and reliable indicator of disease progression and the extent of retinal damage. By matching participants based on DR severity, we aim to ensure meaningful comparisons between male and female groups while minimizing confounding factors related to disease progression.

3. Kindly add in the discussion the advantages of knowing the gender based on the use of AI with retinal images.
How does it help in planning screening / management of DR? These points have to be there to support the study in the discussion section

We appreciate the reviewer for their comment. We would like to clarify that the primary aim of our study was not to predict sex from fundus images but to leverage a model trained for sex classification on retinal images with DR to investigate potential sex differences in the manifestation of DR. By examining how the model attends to different retinal regions in male and female patients, we sought to uncover patterns that might reflect sex-specific variations in the disease's presentation.

As we also highlighted in our response to Reviewer 1 above, understanding these sex differences could have significant implications for the screening and management of DR. For instance, identifying sex-specific manifestations of DR could help improve diagnostic accuracy by tailoring screening methods to account for these differences. This might reduce false positive and false negative rates, ensuring that DR signs unique to one sex are not overlooked or misinterpreted. Additionally, recognizing these variations could guide more personalized approaches to disease management, enabling earlier interventions and better-targeted treatments based on the unique presentation of DR in male and female patients.

We have incorporated these points into the discussion section to further support the study and highlight its clinical relevance:

lines 176-181

This may suggest that, in patients with DR, the retina may exhibit additional sex-specific features beyond what is known to differ in healthy eyes, potentially contributing to the improved predictive power of CNNs trained on eyes with DR. Importantly, the primary aim of this study was not sex classification from fundus images, but rather leveraging the trained model to investigate potential sex differences in the manifestation of DR.

lines 211-220

Differences in the manifestation of DR between males and females have not been extensively investigated, and our findings are among the first to suggest that such differences may exist. Failing to account for these differences in diagnostic approaches may lead to higher false positive and false negative rates. For example, diagnostic algorithms or clinical assessments that consider all symptoms (i.e., the union of symptoms in both sexes) may result in increased false positives, while those that focus only on common symptoms could overlook sex-specific features, increasing false negatives. By identifying and incorporating sex-specific features, diagnostic methods could be refined to reduce both types of errors, enable more accurate

decisions, and mitigate the risks of delayed diagnosis by facilitating earlier detection of DR onset.

---

7. PLOS authors have the option to publish the peer review history of their article (what does this mean?). If published, this will include your full peer review and any attached files.

If you choose “no”, your identity will remain anonymous but your review may still be made public.

Do you want your identity to be public for this peer review? For information about this choice, including consent withdrawal, please see our Privacy Policy.

Reviewer #1: No

Reviewer #2: No
