# Supplementary material for: AI-Assisted identification of sex-specific patterns in diabetic retinopathy using retinal fundus images
Source: PLoS One. 2025 Aug 7;20(8):e0327305. doi: 10.1371/journal.pone.0327305 (PMC12331106; doi:10.1371/journal.pone.0327305)
Supplement: Attachment — Submitted filename: ResponseLetter_R2_IO.pdf [file pone.0327305.s009.pdf]

Dear Dr. Popovic,

Thank you for your editorial work and comments on our manuscript for PLOS ONE. We also thank the two reviewers for their feedback. Below we respond, in a point-by-point fashion, to each comment raised by you and the reviewers. These revisions resulted in several text changes in the manuscript which we have provided here in the letter as well. We hope that, with these revisions, our manuscript is now acceptable to be published in PLOS ONE.

Editor's comments:

- The discussion suggests females may be more prone to ME and males to PDR, but the model only identifies regions of attention, not pathology. There are details comments from this from one of the reviewers that need to be addressed for the manuscript to be accepted.

Thank you for your comment. We agree that the model's saliency maps indicate regions of attention rather than specific pathological features. We have addressed this point in detail in our response to Reviewer #3 (comments 4 and 5) and revised the manuscript accordingly.

- For future research, have you consider validation on an external dataset (e.g., Messidor or IDRiD) to enhance generalizability. This could be ackonwledged in the discussion.

- Though the dataset is stratified by ethnicity, the discussion does not explore whether observed sex-specific differences vary across ethnic groups. This is an opportunity for future research but should be acknowledged.

Thank you for your suggestions. We address both of these points here. We have now added a paragraph to the Discussion about the future directions:

> *"In addition to these directions, future work could benefit from external validation on independent datasets such as Messidor[52] or IDRiD[53] to assess the generalizability of our findings across different populations and imaging conditions. Moreover, although the EyePACS dataset includes individuals from diverse ethnic backgrounds, we did not explore whether sex-related differences in retinal presentation vary across ethnic groups. Understanding how sex and ethnicity may interact in the context of DR could offer valuable insights into disease manifestation and the model's behaviour, and represents an important avenue for future research."*

Reviewer #2: the comments have been addressed satisfactorily by the authors. The revised manuscript reads well. showing sex differences in DME AND PDR

We thank the reviewer for their positive feedback.

Reviewer #3: This manuscript explores the important and timely topic of sex-specific differences in diabetic retinopathy (DR), a complication of diabetes that remains a major cause of vision impairment worldwide. To investigate this, the authors employ an original and creative approach—training convolutional neural networks (CNNs) to classify fundus images as either male or female in patients with DR. The underlying hypothesis is that if DR manifests differently by sex, those differences would be recognized by the CNN as distinguishing features, and subsequently highlighted in saliency (heat) maps.
This manuscript addresses a novel and clinically relevant question using innovative AI-based methodology. The work is technically sound and the results are intriguing.
However, I recommend revisions to ensure consistency in terminology, more cautious interpretation of findings, and a clearer discussion of the study's limitations. With these adjustments, the paper will make a meaningful contribution to the growing field of AI in ophthalmology and precision medicine.

We thank the reviewer for the thoughtful comments. We are glad the reviewer found our work novel and technically sound. Below we address each comment raised by the reviewer.

Comments:
1. Clarification of Sex vs. Gender Terminology: The manuscript would benefit from a more consistent and accurate use of terminology. Sex is a biological classification based on physiological and anatomical characteristics (e.g., chromosomes, hormone profiles, reproductive anatomy), and is indeed relevant to metabolic processes and disease manifestations—as is the focus of this study. In contrast, gender refers to social and cultural roles, personal identity, and lived experience, and is relevant to factors such as access to healthcare, health-seeking behavior, and exposure to stigma or stress—all of which can also impact health outcomes.
However, because the study compares only two groups—male and female—based solely on retinal morphology, and does not include gender identity or sociocultural data, it is more accurate to frame the findings strictly in terms of sex differences. Including both sex and gender as interchangeable terms risks introducing conceptual ambiguity and potentially overextends the claims.
For clarity and scientific precision, I recommend that the manuscript use the term sex consistently throughout and revise or remove references to gender unless supported by appropriate demographic or behavioral data. This will improve the coherence and readability of the text and avoid conflating two distinct dimensions of identity.

We fully agree with the Reviewer's comment. Indeed, our manuscript title refers specifically to sex. We have now revised the manuscript to use the term "sex" consistently throughout the text when referring to our own work (changes are highlighted in the revised manuscript). We have retained the instances of the term "gender" only when citing or discussing studies that explicitly used that terminology.

2. Scope of Analysis and Grouping Strategy: If the intent were to explore both sex and gender differences, the analysis would require more than two comparison groups (e.g., accounting for intersex, transgender, or non-binary individuals), which would significantly increase complexity and demand an entirely different dataset. Since the current study includes only biologically classified males and females, it is appropriate to keep the analysis focused accordingly.

Clarifying this in the introduction and discussion will help anchor the scientific message and prevent misinterpretation.

We appreciate this important clarification. We used a dataset of retinal images to compare male and female patients, based on the 'patient-sex' field recorded in their medical records. The observed differences could potentially reflect biological aspects of sex—such as anatomical or hormonal variations—or may be influenced by broader social factors often associated with gender. However, given that the dataset only includes a binary sex label and lacks detailed sociocultural information, we are not able to distinguish between these possible influences. To reflect this limitation, we refer to these differences as sex-based throughout the manuscript. We have now added a paragraph to the Discussion section to explicitly state that our study is limited to a binary classification based on the 'patient-sex' field in the medical records:

> *"Our analysis was based on the binary 'patient-sex' field in the dataset, which classifies individuals as either male or female. The observed differences may reflect biological aspects of sex, such as hormonal or anatomical variations, or may be influenced by social factors commonly associated with gender, including healthcare access and lifestyle behaviours. However, as our dataset lacks sociocultural and behavioural information, we are unable to disentangle these influences. Future studies incorporating more gender-specific data will be essential to better understand the distinct contributions of sex and gender to DR manifestation."*

3. Absence of Healthy Controls: One notable limitation, which should be more explicitly acknowledged, is the lack of healthy control images in the primary analysis. While the authors cite previous work involving healthy eyes, this study exclusively includes DR-affected eyes. Without healthy controls, it is difficult to determine whether the CNN is identifying sex-specific differences in DR pathology or simply general sex-based anatomical differences that persist regardless of disease. Addressing this limitation more directly in the discussion would enhance the manuscript's rigor.

We thank the Reviewer for this thoughtful comment. We agree that the absence of healthy controls is a limitation. Our EyePACS dataset includes only patients with some degree of DR, which constrained our ability to include healthy controls in the analysis. On the other hand, using another dataset for healthy eyes would introduce a domain shift due to differences in imaging devices, patient populations, and acquisition protocols. Such a shift could affect the model's behavior and interpretation, making it difficult to attribute observed differences directly to DR manifestation. We have now included this point in the Discussion to clarify the scope and limitations of our findings.

> *"Another limitation of our study is the absence of healthy control images in the primary analysis. The EyePACS dataset used in this study includes only individuals with some degree of diabetic retinopathy, preventing direct comparison with healthy eyes. While previous work has investigated sex-based differences in healthy fundus images, we did not include a healthy*

cohort in our model training or evaluation. Incorporating a healthy control group from a different dataset would introduce data distribution shifts due to differences in imaging protocols, equipment, or population characteristics. Such shifts could confound the model's behaviour and make it hard to isolate effects specifically related to DR. Future studies using harmonized datasets that include both healthy and DR-affected eyes from the same source would be valuable in disentangling general sex-based retinal differences from those specific to disease manifestation."

4. Interpretation of AI Output and Causality: The strongest concern lies in the interpretation of the saliency maps and AI-derived findings. Saliency maps indicate the regions of the image that influenced the model's classification decision, but they do not reveal which specific features or pathological changes were used in that decision. Thus, while the CNNs successfully distinguish male from female fundus images with moderate accuracy, this does not confirm the presence of sex-specific disease patterns in DR.
For example, the observed focus on the macular region in female eyes does not necessarily indicate an increased risk of macular edema in women. At most, the findings support that the macula in diabetic females has a distinct appearance compared to diabetic males. To establish a causal link between these imaging features and specific clinical outcomes (e.g., macular edema or progression to PDR), longitudinal studies would be required. This paper does not provide such outcome data or analysis and should avoid implying it does.
Therefore, the current claims regarding differential risk (e.g., "females may be at higher risk for macular edema") are speculative and should be clearly presented as hypotheses for future study, not as conclusions drawn from the present data.

5. Recommendation on Framing and Language: The manuscript would benefit from more cautious phrasing when presenting its conclusions. Statements such as "these findings suggest..." should be tempered with language like "these findings raise the hypothesis that..." or "these findings may indicate...". This will align better with the nature of the data and the exploratory design of the study.

We appreciate these points raised in the comments 4 and 5, and we address them together as they are closely related. We fully agree that saliency maps highlight regions influencing the model's decision but do not specify which underlying features or pathologies are responsible. As the Reviewer notes, while our findings suggest that certain retinal regions—such as the macula in female eyes—are more influential in the model's predictions, we cannot draw conclusions about specific disease patterns or clinical outcomes without additional research. We have revised the language to ensure that our definitive conclusions are limited to identifying the retinal regions where differences between males and females are observed. Statements referring to potential differences in underlying pathological patterns are now clearly framed as hypotheses that stem from these findings. Edited sentences reflecting these changes are provided below and highlighted in the revised manuscript.

Abstract:

"These findings raise the hypothesis that DR may manifest differently by sex,

with women potentially at higher risk for developing ME, as opposed to men who may be at greater risk for PDR."

Results:

"This pattern was consistently observed across the remaining correctly identified images in the test set (S1 and S2 Figs, raising the hypothesis that the information needed for sex classification based on fundus photographs in the presence of DR patients is located in different regions of the retina."

Discussions:

"Based on this, we hypothesize that sex differences in DR-affected eyes may be reflected in the model's attention to different retinal regions, though the specific features driving this behavior remain unclear."

"Our findings suggest that DR may manifest differently in males and females…"
