# Supplementary material for: AI-Assisted identification of sex-specific patterns in diabetic retinopathy using retinal fundus images
Source: PLoS One. 2025 Aug 7;20(8):e0327305. doi: 10.1371/journal.pone.0327305 (PMC12331106; doi:10.1371/journal.pone.0327305)
Supplement: Attachment — Submitted filename: PONE-D-24-39869R1.docx [file pone.0327305.s007.docx]

**Dear Editor,**

I have reviewed the manuscript titled "AI-Assisted Identification of Sex-Specific Patterns in Diabetic Retinopathy Using Retinal Fundus Images." While the study explores a novel angle by focusing on sex-specific patterns in diabetic retinopathy, there are several areas that need significant improvement to enhance its scientific contribution and clarity:

- The rationale for identifying sex-specific patterns in diabetic retinopathy is not well-justified. The authors should provide a stronger explanation of the clinical relevance and potential impact of their findings.
- It is unclear how this approach contributes to improving diagnosis or treatment strategies compared to traditional methods.
- The dataset used for training and validation is not described in detail. Critical information such as sample size, class balance, preprocessing steps, and demographic distribution is missing.
- Include more recent references that are relevant to the topic, such as
- Al-hazaimeh, Obaida M., Ashraf A. Abu-Ein, Nedal M. Tahat, Ma'moun A. Al-Smadi, and Malek M. Al-Nawashi. "Combining Artificial Intelligence and Image Processing for Diagnosing Diabetic Retinopathy in Retinal Fundus Images." *International Journal of Online & Biomedical Engineering* 18, no. 13 (2022).
- Gharaibeh, Nasr, Obaida M. Al-Hazaimeh, Bassam Al-Naami, and Khalid MO Nahar. "An effective image processing method for detection of diabetic retinopathy diseases from retinal fundus images." *International Journal of Signal and Imaging Systems Engineering* 11, no. 4 (2018): 206-216.

Thank you for allowing me to review this manuscript. Please do not hesitate to reach out for further clarification or additional feedback.

**Best regards,**
